# Potassium bicarbonate and D-ribose effects on A72 canine and HTB-126 human cancer cell line proliferation in vitro

**DOI:** 10.1186/1475-2867-11-30

**Published:** 2011-08-22

**Authors:** Simonetta Croci, Luca Bruni, Simona Bussolati, Marianna Castaldo, Maurizio Dondi

**Affiliations:** 1 Dipartimento di Sanità Pubblica, Sezione di Fisica, University of Parma Via Volturno 39, Parma, Italy; 2 INBB (Istituto Nazionale Biostrutture e Biosistemi) Via delle medaglie d'Oro 305, Roma, Italy; 3 Valsè Pantellini Foundation, Calle Cervantes, 16/4 izda, Oviedo Asturia, Spain; 4 Dipartimento di Produzioni Animali, Biotecnologie Veterinarie, Qualità e Sicurezza degli Alimenti-Sezione di Fisiologia Veterinaria, University of Parma, Via del taglio 10, Parma, Italy; 5 Dipartimento di Salute Animale, Sezione di Clinica Medica Veterinaria, University of Parma, Via del taglio 10, Parma, Italy

**Keywords:** A72 canine cancer cell line, HTB-126 human cancer cell line, potassium bicarbonate, D-ribose, MTT assay, cancer, cell proliferation

## Abstract

**Background:**

The synergic action of KHCO_3 _and D-ribose is tested on A72 and HTB-126 cell lines proliferation using K:D-Rib solution. Altered Na^+^/K^+ ^ATPase expression and activity were shown in patients with cancer. Studies in human epithelial-derived malignancies indicate that K^+ ^depletion also occurs, contributing to the increased intracellular Na^+^/K^+ ^ratio [1]. D-ribose transformed to piruvate, enters into the Krebs's cycle and has a key role on energetic metabolism. The up-regulation of glycolysis in tumor cells is already well known and it is the rationale of F^18^-FDG PET diagnostic technique. D-ribose is synthesized by the non-oxidative transketolase PPP reaction.

**Results:**

Results with different K:D-Rib concentrations show that MTT salt interferes with K:D-Rib solution and therefore this method is not reliable. The UV/VIS measurements show that K:D-Rib solutions reduce MTT salt to formazan in absence of cells. Cell proliferation has then been evaluated analysing the digital photos of the Giemsa stained cells with MCID™ software. At 5 mM K:D-Rib concentration, the cell growth arrests between 48 h and 72 h; in fact the cell number after 48 h is around the same with respect to the control after 72 h. In case of HTB-126 human cancer cells, the growth rate was valuated counting the splitting times during 48 days: control cells were split sixteen times while 5 mM treated cells eleven times. Most relevant, the clonogenic assay shows that nine colonies are formed in the control cells while only one is formed in the 5 mM and none in 10 mM treated cells.

**Conclusions:**

The K:D-Rib solution has an antioxidant behaviour also at low concentrations. Incubation with 5 mM K:D-Rib solution on A72 cells shows a cytostatic effect at 5 mM, but it needs more than 24 h of incubation time to evidence this effect on cell proliferation. At the same concentration on human HTB-126 cells, K:D-Rib solution shows a clear replication slowing but the cytostatic effect at 10 mM K:D-Rib solution only. Results on A72 cells indicate the K^+ ^uptake could be determinant either to arrest or to slow down cell growth.

## Introduction

Cancer is still one of the major causes of death despite many years of study and many different research approaches. The theory of Cone [2] proposes that sustained depolarization of cell membrane is involved in the regulation and control of cell division during both normal and cancerous growth of tissues. Using X-Ray microanalytic method [3] the correlation between the high Na^+ ^content and the proliferative cell capacity has been proved in different cell types [4,5]. The scope of the present work is to assess the effects of potassium hydrogen carbonate and D-ribose water solution (K:D-Rib) on A72 canine and HTB-126 human cancer cell proliferation. Our previous study [6] showed the strong antioxidant effect of potassium ascorbate (KAsc) on red blood cell oxidation [7,8]. That work [6] focused on potassium ascorbate which seems to act as a carrier of K^+ ^inside the cells equilibrating the K^+ ^inner concentration. Potassium is essential for cell life and it is involved in many different cell pathways. Under physiological condition, K^+ ^has intracellular concentration of about 150 mM and an extracellular concentration of about 5 mM. Intracellular homeostasis for Na^+ ^and K^+ ^is no longer tightly regulated in cancer cells. An alteration of Na^+^/K^+ ^ATPase expression and activity has been shown in patients with cancer [9,10]. Epidemiologic evidence suggests that a high K^+ ^intake inhibits cancer development, and a high Na^+ ^intake increases the incidence of gastrointestinal malignancies. Studies in human epithelial-derived malignancies indicate that K^+ ^depletion also occurs, contributing to the increased intracellular Na^+^/K^+ ^ratio [1]. Na^+^/K^+ ^ATPase uses energy from the hydrolysis of ATP to drive K^+ ^into cells in exchange for Na^+^, which in turn, provides the driving force for the transport of other solutes, notably amino acids, sugars, and phosphates [10]. It has been found that K^+ ^is essential to fold and to stabilize G-quadruplex [11]. Agents that stabilize G-quadruplexes can act as anti-tumour agents, so physiological K^+ ^concentration is responsible for normal cell behaviour [12,13]. Despite these findings, many questions on the key role of K^+ ^are still not completely clarified, in fact K^+ ^has a significant role also on apoptotic events [14].

D-ribose, is a penta-sugar precursor of some amino acids like glutamate, histidine, proline and arginine. D-ribose transformed to piruvate, enters into the Krebs's cycle and has a key role on energetic metabolism; it is also involved in glycogen synthesis. The up-regulation of glycolysis in tumour cells is already well known and it is the rationale of F^18^-FDG PET diagnostic technique [15]. Glucose could be used for energy production through glycolysis, energy storage through glycogen synthesis and for intracellular process through the pentose phosphate pathways (PPP) [3]. Constitutive up-regulation of glycolysis is also observed even in the presence of adequate oxygen supplies (aerobic glycolysis): a phenomenon first noted by Warburg effect [16]. D-ribose can be directly synthesized by the non-oxidative transketolase PPP reaction transferring a keto group to glyceraldeyde 3-phoshate from fructose 6-phosphate. Both these substrates are intermediate products of glycolysis.

Because of all this evidence, we started testing the effect of K:D-Rib on A72 cell line proliferation at different concentrations: 5 mM, 0.5 mM, and 0.05 mM. The cell growth is evaluated using MCDI™ software. We move on testing K:D-Rib solution on HTB-126 human cancer cell line. The effect of 5 mM K:D-Rib solution on proliferation and replication capability was valuated with clonogenic assay and counting the number of splitting during 48 days. Preliminary experiments are reported showing the interaction between K:D-Rib and 3-[4,5- dimethylthiazolyl-2]-2,5-diphenyltetrazolium bromide (MTT), which is commonly used to test the cell proliferation and viability.

## Results

**Figure 1:**
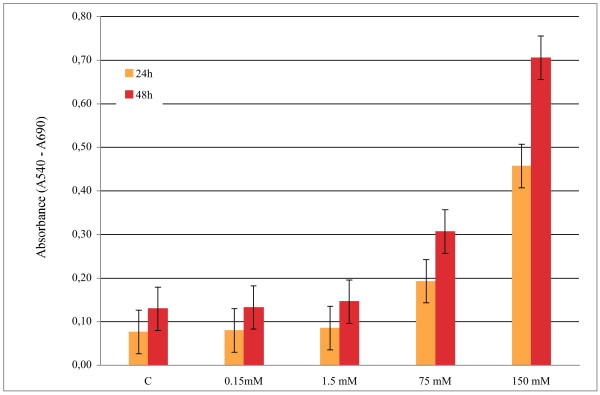
**MTT absorbance values of A72 cell line cultures**. Absorbance values of A72 cell line culture after 24 h and 48 h after 24 h and 48 h, without K:D-Rib treatment - C - and at different K:D-Rib concentrations - 0.15 mM - 1.5 mM - 75 mM - 150 mM determined by MTT assay. A72 cells (2 × 10^4 ^cells/well) were sown and cultured at standard conditions (5% CO_2_, 37°C and humidified atmosphere).

The MTT proliferation assay was performed testing the K:D-Rib concentrations from 0.15 mM to 150 mM, according to methods. Absorbance data at 540 nm with respect to 690 nm are reported in Figure 1. Values at 0.15 mM and 1.5 mM K:D-Rib concentrations indicate no significant differences between control and treated cells.

At higher K:D-Rib concentrations (75 mM and 150 mM) absorbance soars in the treated samples with respect to the control, both at 24 h and 48 h.

**Figure 2:**
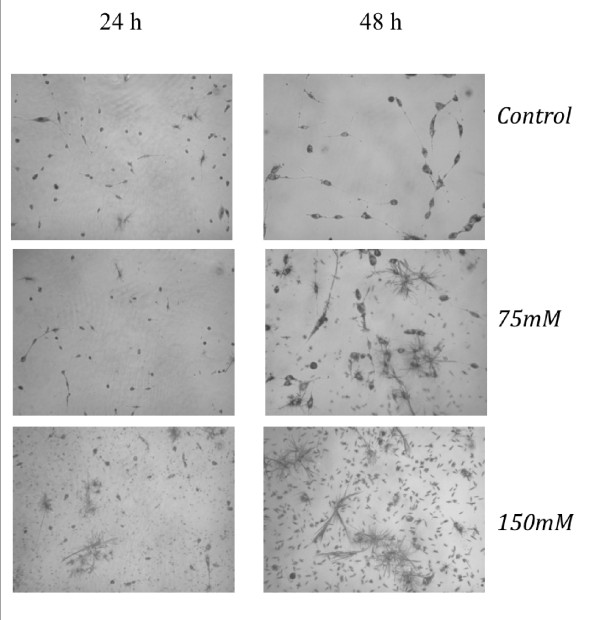
**Microscope images of A72 cells with MTT salt**. A72 cell images: control, 75 mM K:D-Rib and 150 mM K:D-Rib after 24 h and 48 h of incubation. The pictures were taken just after the MTT assay measurements whose absorbance values, at 75 mM and at 150 mM K:D-Rib concentrations are reported in Figure 1.

On the other hand, the pictures taken with the digital microscope camera display fewer cell in the 75 mM sample at 24 h as shown in Figure 2. In this case cells exhibit morphological damages and they are no more viable, both at 48 h (75 mM K:D-Rib) and at 24 h (150 mM K:D-Rib). It seems that at these K:D-Rib concentrations the MTT salt interacts with the K:D-Rib solution.

**Figure 3:**
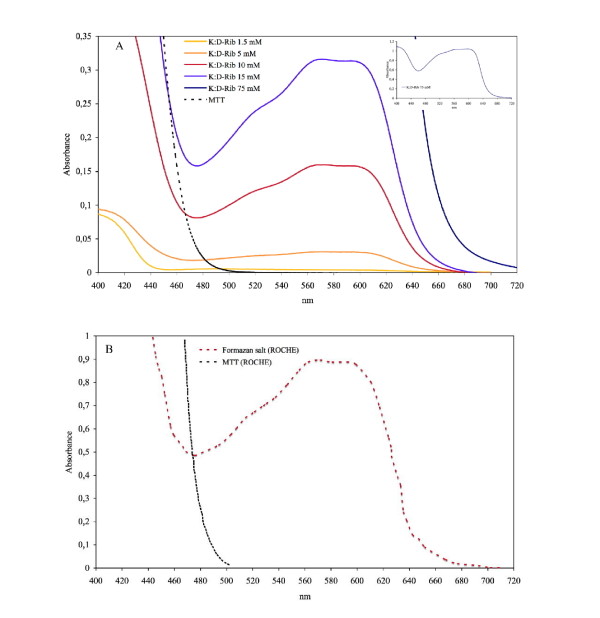
**UV/VIS absorbance spectra of MTT salt with and without K:D-Rib solution**. A, black dashed line: MTT - SDS UV/VIS spectrum; coloured solid lines: MTT - K:D-Rib interaction product UV/VIS spectra after solubilization obtained subtracting the MTT- SDS spectrum from raw data. Different K:D-Rib concentrations, 1.5 mM, 5 mM, 10 mM, 15 mM and 75 mM (inset) were used. B, black line: MTT salt UV/VIS spectrum; red line: formazan salt after solubilization (ROCHE data sheet).

To this purpose we analyzed the UV/VIS absorption spectrum to highlight the products of a MTT - K:D-Rib reaction. Samples with MTT salt were prepared, according to methods, with different K:D-Rib concentrations: 150 mM (S150), 75 mM (S75), 15 mM (S15), 10 mM (S10) 5 mM (S5) and 1.5 mM (S1.5). The S150 sample did not show a good solubilization and some crystals were present in the solution, therefore we thought the UV/VIS spectrum for this sample had to be discarded. The spectrum of a MTT solution with SDS is also reported in Figure 3A (dashed line). Because the MTT salt was not completely reduced by K:D-Rib solution, to obtain MTT - K:D-Rib interaction product spectrum we cleaned the raw spectra by subtracting the MTT-SDS solution spectrum from the S75, S15, S10, S5 and S1.5 spectra, being the K:D-Rib solution absorption neglible between 400 nm and 720 nm.

In the inset of Figure 3A, 75 mM K:D-Rib sample spectrum is shown. Figure 3B displays the spectra of MTT salt and of formazan after solubilization as shown by ROCHE data sheet of Cell Proliferation Kit I (MTT). Comparing the formazan spectrum of Figure 3B (dashed line) with spectra of Figure 3A (solid lines) it is clear that K:D-Rib interacts with MTT producing formazan salt. It is also evident that the amount of formazan is proportional to K:D-Rib concentration.

**Figure 4:**
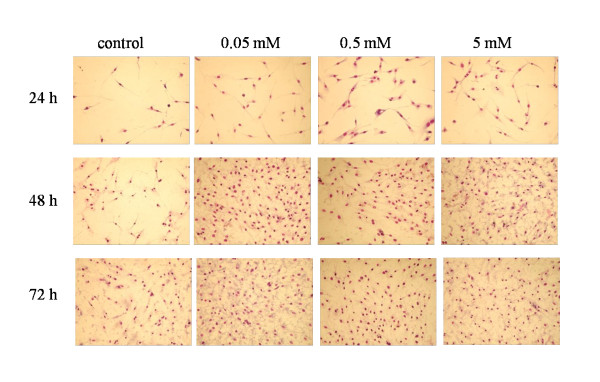
**A72 cell images representative of one region for each cell plate**. Images of A72 cells stained with Giemsa stain solution: nucleus is brown red and cytoplasm is light red. The control cells and cells with three different K:D-Rib concentrations (0.05 mM, 0.5 mM and 5 mM) were tested at three different time points (24 h, 48 h and 72 h). A72 cells (4 × 10^4 ^cells/ml) were sown and cultured at standard conditions (5% CO_2_, 37°C and humidified atmosphere).

Because all this evidence, the cell proliferation and morphology were then evaluated by means of the digital photos analysed with MCID™ program. The treated cells, incubated with 5 mM, 0.5 mM and 0.05 mM K:D-Rib were then fixed and stained, as stated in methods. The effects were evaluated after 24 h, 48 h and 72 h from sowing. To prove accuracy of the analysis we define in each stained plate six different regions and we took separate pictures of all of them. Using the MCID™ program one is able to identify the stained nucleus and therefore to count the number of cells in each picture. An example of one region for each cell plate is presented in Figure 4.

**Figure 5:**
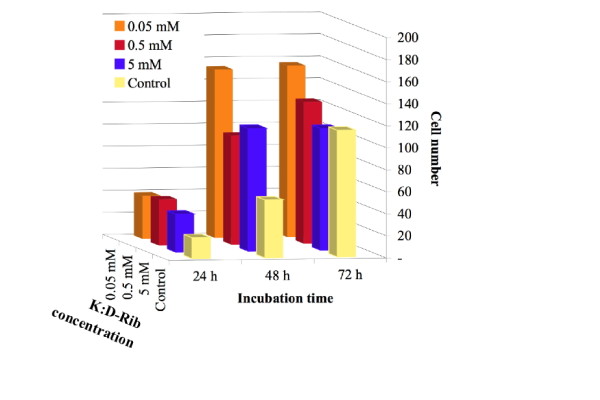
**Cell number at different incubation time and at different K:D-Rib concentrations**. Bars are the cell number averages of the six pictures of a given cell plate. Bars are relative to control cells and to K:D-Rib treated cells (0.05 mM, 0.5 mM, 5 mM) at three different time points (24 h, 48 h and 72 h).

Every picture was analysed by MCDI™ software and the overall count of a plate was computed averaging together the results for the six different pictures of a plate. The data reported in Figure 5 are the cell number averages of the six pictures of a given cell plate. The graph relates the cell number with concentration and incubation time. It is clear that cells treated with 0.05 mM, 0.5 mM and 5 mM K:D-Rib grow faster than control both after 24 h and after 48 h, but a different kinetic shows up after 72 h. At 0.05 mM K:D-Rib concentration after 48 h from sowing the cell number is three times higher than control. There is no significant growth between 48 h and 72 h. Conversely the 0.5 mM K:D-Rib concentration has a completely different effect: cells grow markedly slower than cells treated with 0.05 mM K:D-Rib both at 48 h and 72 h. At 5 mM (highest K:D-Rib concentration tested on A72) the cell number after 48 h is around the same with respect to the control after 72 h. In the case of 5 mM K:D-Rib treated cells, growth arrests between 48 h and 72 h. Due to the previous results, the 5 mM K:D-Rib concentration was also tested on HTB-126 human cancer cells.

In order to assess the effect of 5 mM K:D-Rib on cell proliferation we carried out a growth rate estimation by observing our sown cells over a 48 day period of time. When the sown cells reached the 90% - 100% confluence, they were split 1:2. After 48 days control was split 16 times while the 5 mM K:D-Rib treated cells 11 times.

**Figure 6:**
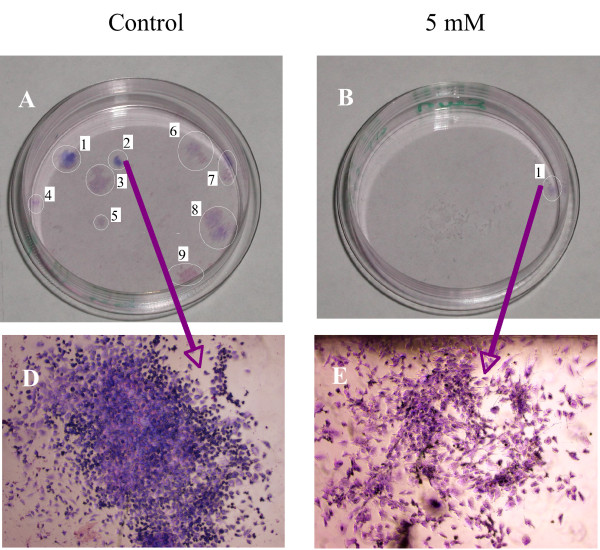
**HTB-126 clonogenic assay**. A, Petri dish with the 9 highlighted HTB-126 control cell colonies; D, colony No. 2 of the control Petri dish; C, Petri dish with 1 highlighted of HTB-126 5 mM K:D-Rib treated cells colony; E, colony No. 1 of the 5 mM K:D-Rib treated Petri dish.

To test the cell capability to survive and replicate following the incubation with 5 mM and 10 mM K:D-Rib solution, clonogenic assay was performed. A colony is defined as a cluster of at least 50 cells. We count nine colonies in the control sample as is shown in Figure 6A. In Figure 6C the picture of a representative colony of the control sample is reported. In the case of the 5 mM K:D-Rib treated cells, only one colony is formed as reported in Figure 6B and 6D, but at 10 mM K:D-Rib there was no colony.

## Discussion and conclusions

The MTT assay data at high K:D-Rib concentrations, 75 mM and 150 mM, suggest an enhancement of the cell proliferation. According to these absorbance values, cells should be viable and proliferative at any K:D-Rib concentration. But as one can see from the results shown in Figure 2, the previous conclusions clash with the observation of the morphological damages brought about the treated cells. These results are not so surprising, in fact the samples with 75 mM and 150 mM K:D-Rib solutions have a K^+ ^concentration which is between forty and ninety times higher than physiological extracellular concentration (5 mM). The cell death could be a consequence of the culture medium osmolality. What actually is more surprising is the non-reliability of the MTT test. These results, together with results obtained from UV/VIS spectroscopy, prove the interaction of MTT with the K:D-Rib solution with the following consequences. The K:D-Rib solution reduces MTT salt to formazan with a behaviour similar to ascorbic acid behaviour. Spontaneously the ascorbic acid reduces MTT salt to a formazan as it reduces many compounds such as H_2_O_2 _and cytochrom *c *[17]. This anti-oxidant behaviour of K:D-Ribose solution is evident also at low concentrations of about 5 mM. Because the absorbance results of the MTT assay provide erroneous information about cell viability, the cells were then stained and counted with help of the MCID™ program. K:D-Rib solution has two important components D-ribose and potassium, actually the K^+ ^source is the potassium hydrogen carbonate. As is shown in Figure 5, the cell population of all this treated plates is not significantly different after 24 h of incubation time but it is higher than control. One can deduce that D-ribose enters into the cell [18] and it is metabolized as carbon source increasing cell growth and consequently the cell number. Highest growth enhancement occurs at 0.05 mM K:D-Rib between 24 h and 48 h. In this case, the K^+ ^concentration is 0.15 mM, thirty times lower than the extracellular concentration (~5 mM), not enough to change any ion balance, thus the population increase has to be attributed to D-ribose sugar. K^+ ^may tend to rebalance its intracellular concentration and re-establish the normal cellular functions, but only the effect of D-ribose is evident at low K^+ ^intracellular concentration. At 0.5 mM K:D-Rib, the cell growth is slowed down with respect to the 0.05 mM K:D-Rib treated cells, but at this concentration there is no cell growth stop. In this case, in accordance with the previous interpretation, the K^+ ^begins to show its effect, but it is not enough to stop the increase of cell population. Indeed, at 5 mM K:D-Rib concentration, the solution has a cytostatic effect. Cell growth slows down with respect to 0.05 mM after 48 h and to the 0.5 mM. In addition they stop their growth between 48 h and 72 h from sowing.

Firstly from UV/VIS results, we can conclude that the K:D-Rib has a antioxidant behaviour also at concentration of about 5 mM. On A72 cancer cells K:D-Rib, at any tested concentration, needs more than 24 h of incubation time to show effects on cell proliferation, but at 5 mM it shows a cytostatic effect. K^+ ^uptake seems to be determinant either to arrest or to slow down cell growth. This effect at 5 mM cannot be attributed to an osmolality effect since the K^+ ^concentration is of same order of magnitude of the physiological concentration. At 0.05 mM K:D-Rib concentration the K^+ ^concentration is thirty times lower than physiological extra cellular one; only the effect of D-ribose is visible, and cell are growing. Starting with higher K^+ ^concentration we see a marked slow down in the cell growth. We suggest that D-ribose is acting as a "Trojan horse" that is carrying K^+ ^inside the cells. These preliminary results are of fundamental importance and they have been the starting point for studying the K:D-Rib solution effect on human cancer cells. Because we had the most relevant results with 5 mM K:D-Rib concentration, we started with the same concentration on HTB-126 human breast cancer cells. As reported in the results section an established slowing of cell growth rate was demonstrated during 48 days treatment: the control cells were split every 2.7 days on average while the 5 mM K:D-Rib treated cells were split every 4 days on average, for a total of 16 and 11 times respectively. This result is confirmed by the clonogenic assay where nine colonies were formed in control with respect to only one in the 5 mM K:D-Rib treated sample. These results on a human breast cancer cell line at 5 mM K:D-Rib show a similar effect than on A72 cell line, in fact the cell replication is strongly reduced although not completely blocked. At this concentration we did not see cytostatic effect on HTB-126 cells but the cytostatic effect was evident at 10 mM K:D-Rib. Actually it is not surprising that different cell system have a different dose-effect response.

In the next step we will continue to test the K:D-Rib on human cancer cells and in the meanwhile we will try to have a better understanding on the cellular mechanisms that are involved in this cytostatic behaviour focusing on K^+ ^and D-ribose role.

Finally we also suggest a careful use of MTT assay test when the interactions between cells with other system are tested [19,20].

## Methods

### Cells and culture conditions

A72 adherent canine tumour cell line was obtained from American Type Culture Collection (Manassas, VA, USA). A72 cells were maintained in high glucose Dulbecco's Modified Eagle's Medium (Sigma Aldrich) supplemented by: 10% Fetal Calf Serum (Sigma Aldrich) and 1% L-Glutamine (Sigma Aldrich), 1% Penicillin-Streptomycin (Sigma Aldrich). A72 cell line was incubated at 37°C in humidified atmosphere with 5% CO_2_.

HTB-126 adherent human breast cancer cell line was obtained from American Type Culture Collection (Manassas, VA, USA). HTB-126 cells were maintained in low glucose Dulbecco's Modified Eagle's Medium (Lonza) supplemented by: 10% Fetal Calf Serum (Lonza), 1% L-Glutamine (Lonza), and 1% Penicillin-Streptomycin (Lonza). HTB-126 cell line was incubated at 37°C in humidified atmosphere with 5% CO_2_.

### Chemicals

Giemsa stain modified solution was bought from Fluka. MTT yellow salt (3-[4,5-dimethylthiazolyl-2]-2,5-diphenyltetrazolium bromide), paraformaldehyde powder and sodium dodecilsulfate (SDS) were purchased from Sigma Aldrich. HCl was bought from MERCK.

### Drugs

D-ribose was bought from Sigma-Aldrich and potassium hydrogen carbonate from BDH. 1 M K:D-Rib solution is obtained by combining 0.15 mg of D-ribose and 0.3 mg of potassium hydrogen carbonate in distilled water; then waiting for the CO_2 _to dissipate. The concentrations desired were reached diluting the stock solution with distilled water.

### MTT proliferation assay

The MTT *assay *was employed to valuate the K:D-Rib effects on cell proliferation and viability. The K:D-Rib tested concentrations were: 0.15 mM, 1.5 mM, 75 mM and 150 mM. A72 cell line was sown at 1000 cells/100 μl concentration into a 96-well plate. MTT salt is reduced to formazan in the metabolic active cells by dehydrogenase to form NADH and NADPH. The cell reduction product, called formazan displays a purple colour. It can be quantified spectroscopically after solubilization. From each well, 100 μl of surnatant were discarded. Then 20 μl of MTT (reconstituted powder in PBS 1X at 5 mg/ml) were added to each well. The cells with MTT solution were incubated for 4 h at 37°C. Then 100 μl of solubilization buffer (SDS 10% in HCL 0.01 N) were added to the cells and they were incubated at 37°C overnight. At the end of this incubation time, the assayed plates were read by Spectra Shell microplate reader, at the wavelength of 540 nm respects to 690 nm. The absorbance was measured at 24 h and 48 h after sowing. Absorbance is proportional to the number of viable cells.

### Optical measurements

A UV/VIS Varian spectrometer was used from 400 nm to 720 nm to follow the MTT salt reaction with K:D-Rib solution. In case of K:D-Rib the tested concentrations were: 150 mM, 75 mM, 15 mM, 10 mM, 5 mM, 1.5 mM. 200 μl of MTT solution were added in 2 ml K:D-Rib solution. The samples were measured, similarly to the procedure done with the cells, after an incubation time of 4 h at 37°C, followed by the addition of 2 ml of solubilization solution (sodium dodecilsulfate SDS 10%) left overnight.

### Microscope cell counting

The tested K:D-Rib concentrations were: 0.05 mM, 0.5 mM and 5 mM. The counts were performed at 24 h, 48 h and 72 h from sowing. 40000 cells/ml were sown into a 4-well plate. The cells were fixed at air flux of the biological cape, at every time point for one hour. After that, the cells were stained by Giemsa stain solution and photographed by microscope digital camera. Cell counting was done analyzing the cell images with MCID™ software.

### Splitting number

The tested K:D-Rib concentration was 5 mM. HTB-126 cell line was sown at 10000 cells/ml into 35 mm Petri dish. When the cells were approximately 90 - 100% confluent they were split 1:2. This procedure continued for 48 days. The splitting number is reported both for control cells and for 5 mM K:D-Rib treated cells.

### Clonogenic assay

24 h before treatment, HTB-126 cells were sown at the concentration of 25 cells/ml in a 35 mm Petri dish, waiting for the cells adhesion. The tested K:D-Rib concentrations were 5 mM and 10 mM; the treatment lasted for tree weeks. The cell medium was changed once a week. After tree weeks colonies were fixed with paraformaldehyde (4.0% v/v), stained with Giemsa solution (5.0% v/v) and counted using an inverted microscope. A colony is defined to consist of at least 50 cells.
